# Age-related changes in neuroinflammation and prepulse inhibition in offspring of rats treated with Poly I:C in early gestation

**DOI:** 10.1186/s12993-019-0154-2

**Published:** 2019-03-05

**Authors:** Shuang Ding, Yunqing Hu, Binbin Luo, Yaqi Cai, Keke Hao, Yongfeng Yang, Yan Zhang, Xiujuan Wang, Minli Ding, Hongxing Zhang, Wenqiang Li, Luxian Lv

**Affiliations:** 1 Henan Mental Hospital, The Second Affiliated Hospital of Xinxiang Medical University, No. 388, Jianshe Middle Road, Xinxiang, 453002 Henan People’s Republic of China; 2 Henan Key Lab of Biological Psychiatry of Xinxiang Medical University, Xinxiang, China; 3 International Joint Research Laboratory for Psychiatry and Neuroscience of Henan, Xinxiang, China

**Keywords:** Maternal immune activation, Schizophrenia, Poly I:C, Microglia, Astrocyte

## Abstract

**Background:**

Maternal immune activation (MIA) during gestation can increase the later risk of schizophrenia in adult offspring. Neuroinflammation is believed to underlie this process. Postmortem brain studies have found changes in the neuroimmune systems of patients with schizophrenia. However, little is known about the dynamic changes in cerebral inflammation and behavior during the course of the disease.

**Methods:**

Here, the prepulse inhibition (PPI) test was conducted in adolescent and adult Sprague–Dawley rats prenatally challenged with polyriboinosinic–polyribocytidylic acid (Poly I:C) on gestational day 9 to determine the behavioral trajectory triggered by early exposure to Poly I:C. Brain immune changes were determined in the prefrontal cortex (PFC) and hippocampus (HC) at both ages. The status of the microglia and astrocytes was determined with immunohistochemical staining. The levels of IL-6, IL-1β, and TNF-α in both brain regions were evaluated with enzyme-linked immunosorbent assays.

**Results:**

Disrupted PPI, the core phenotype of schizophrenia, only emerged in adulthood. Behavioral changes during puberty and adulthood were both accompanied by the activation of microglia (PFC and HC). Astrocytes were only activated at PN60. The levels of proinflammatory cytokines (IL-1β, IL-6, and TNF-α) in the offspring of the Poly I:C-exposed mothers differed with brain region and time, with more cytokines elevated during periadolescence than during adulthood.

**Conclusions:**

Our findings indicate that immune activation emerged before symptom manifestation in the offspring of MIA rats. We conclude that early prenatal Poly I:C challenge can lead to age-related behavioral and neuroinflammatory changes. These data provide new insight into the neuroinflammatory and neuropathological mechanisms underlying the development of schizophrenia. They also suggest that periadolescence could be more important than adulthood in the prevention and treatment of schizophrenia.

## Background

Schizophrenia is a chronic and devastating disorder affecting ~ 1% of the world population [1]. The pathogenesis of schizophrenia involves an interplay between genetic and environmental factors. Although the genetic contribution is large [2], the importance of the environment in the development of this disease is increasingly recognized.

Epidemiological surveys have indicated that prenatal maternal infections with various infectious agents are risk factors for the development of schizophrenia in the adult offspring [3–6]. Examples include influenza virus, rubella, cytomegalovirus, Herpes simplex virus 2, *Chlamydia*, *Toxoplasma gondii*, and Borna disease virus. Interestingly, diverse inflammatory events can have similar consequences on the brain. Therefore, it has been postulated that the effects on behavior and brain function may depend on the immune responses and factors that mediate these processes, such as cytokines and immune cells, rather than specific pathogens. Researches in animal models of maternal immune activation (MIA) suggest that synthetic viruses or bacterial analogues can also lead to behavioral changes related to schizophrenia, further supporting the notion that inflammation is a key player in the pathophysiology of this disorder [7].

Today, there is renewed interest in brain inflammatory changes and the key roles they play in the pathophysiological mechanisms of schizophrenia [8]. Emerging evidence indicates that neuroinflammation is related to schizophrenia [9–11]. For instance, the expression of immune-related genes is increased in the prefrontal cortices of schizophrenic patients [12], and there is also evidence that cytokine levels are abnormal in the cerebrospinal fluid and specific brain areas of patients with schizophrenia [13–16]. The immune processes of the central nervous system (CNS) are complex and are still only partly understood. Microglia and astrocytes are the major immune cells in the brain [17, 18], and postmortem studies have shown increased cerebral microgliosis and astrogliosis in schizophrenic patients [19–23], although not consistently [24–27]. In fact, microglia are the main resident immune cell population in the CNS. When the environment changes, microglia are usually the first to alter in morphology and function to react to those challenges [28], and their activation is believed to be linked to the pathophysiology of schizophrenia [29]. Like microglia, astrocytes also have immunological functions, although less importance has been ascribed to them as to those of microglia. Astrocytes, the largest and most numerous glial cells in the CNS, also produce pro- and anti-inflammatory cytokines, including interleukin (IL)-1, IL-6, tumor necrosis factor α (TNF-α), transforming growth factor β (TGF-β), interferon (IFN)-α, and IFN-β, that participate in the innate and adaptive immune processes in the brain [30, 31]. Although many studies have confirmed the involvement of microglia in schizophrenia, the role of astrocytes remains very controversial.

To better understand the pathogenic mechanism of schizophrenia, animal models of MIA have been established [32, 33]. Polyriboinosinic–polyribocytidylic acid (poly[I:C]), a synthetic double-stranded RNA, mimics viral infection by activating toll-like receptor 3 [32]. Numerous studies have shown that the offspring of pregnant dams treated with Poly I:C show a battery of schizophrenia-like behaviors and neuroimmunological abnormalities. Among these are defects in the prepulse inhibition (PPI) and cognition [34], impairment of locomotor activities, deficits in learning skills, the dysregulation of neurotransmission [7, 35], and brain morphological abnormalities [36, 37]. Therefore, the Poly I:C MIA model has become a powerful tool in the investigation of the progressive nature of schizophrenia. This model has also been used to develop therapeutic and preventive strategies to halt disease progression [38], which indirectly indicate possible mechanisms underlying disease. In one study, Osborne et al. have shown that CBD, a component with anti-inflammatory property, can attenuate cognitive deficits and the social interaction induced by prenatal Poly I:C infection [34]. Another experimental research using Poly I:C MIA model has suggested that minocycline treatment can prevent the behavioral aberrations and microglial changes associated with schizophrenia [39]. Besides, the administration of cyclo-oxygenase 2 inhibitors, an anti-inflammatory medication, in the early stages of schizophrenia has had beneficial effects [40]. Taken together, these studies suggest the potential utility of treating inflammation in the asymptomatic period of schizophrenia to prevent disease development.

For a long time, the mid-late prenatal period was thought to be the only risk window in which schizophrenia developed based on the initial human epidemiological studies [6], and many researchers have examined this with the rodent equivalent, gestational day (GD)15 or GD17 as the date for Poly I:C stimulation [34–36]. However, several studies have reported that early prenatal insult also results in psychiatric diseases [41], an important but long-ignored fact. Several experimental studies pinpointed the precise stage of pregnancy at which infection may cause different behavioral phenotypes [42]. MIA at GD9 specifically induces abnormal behaviors, which are related to the positive symptoms of schizophrenia, while Poly I:C exposure on GD17 could cause mostly negative symptoms [43]. However, there are limited data regarding the abnormalities in rat after early prenatal MIA, especially alternations of astrocyte as mentioned above.

Therefore, we tested the hypothesis that MIA induced by exposure to Poly I:C at GD9 induces age-related behavioral and neuroinflammatory changes in the offspring.

## Methods

### Animals

Eight-week-old male and female Sprague–Dawley rats were obtained from a specific-pathogen-free breeding colony at the Experimental Animal Center of Zhengzhou University (Zhengzhou, China). The animals came from multiple litters. Littermates of the same sex were caged together, with 3–4 per cage. Breeding began after 2 weeks of acclimation to the new animal holding room. The procedures for breeding and for the verification of pregnancy have been described by Meyer [44]. The rats were housed individually in ventilated plastic cages at 22 ± 2 °C and 50 ± 10% relative humidity with a constant day–night cycle (light: 08:00–20:00 h). Food and tap water were available ad libitum. The Animal Care and Use Committee of the Henan Key Laboratory of Biological Psychiatry (Xinxiang, China) approved the use of the rats and the experimental protocols in this study.

### MIA during pregnancy

The timed pregnant mice were injected intravenously with 0.1 mL of saline or 10 mg/kg Poly I:C (Sigma-Aldrich, St. Louis, State of Missouri, USA) on GD9. All the animals were immediately returned to their home cages after injection. On postnatal day (PN) 21, both groups of pups were weaned, then housed 3–4 to a cage according to sex and litter. Half of each group was maintained undisturbed until 6 weeks of age (PN40), and the rest until 8 weeks of age (PN60), which correspond to periadolescence and young adulthood, respectively, in humans [45].

### Behavioral testing

A total of 20 animals (11 males for cases; 9 males for controls) and 21 animals (13 males for cases; 8 males for controls) were randomly selected at PN40 (adolescent stage) and PN60 (young adult stage), respectively, for behavioral tests to evaluate the manifestations of schizophrenia-like behavior. All behavioral tests were performed between 08:00 and 18:00 h.

#### PPI test

PPI was tested in four sound-attenuated chambers. All test sessions were performed in a single-chamber startle apparatus (QMC-I, Kunming Institute of Zoology, Chinese Academy of Sciences, China). After the mice were allowed to adapt for 5 min, the white noise was set to 70 decibels (dB) for 10 pretests, and then rats went to the test phase, as previously described [46]. The experiment consisted of 40 rounds of stimulation, which began with a delay of ~ 50 ms, followed by a 20 ms pulse stimulation with white noise (75, 80, or 85 dB), followed by a 100 ms delay, and then a 40 ms stimulation of the startle reflex with white noise of 120 dB. The last was 290 ms to record time. Each trial was completed in 500 ms, and the average mutation interval was 15 s. In this experiment, eight different types of stimuli were supplied: pulse stimulation, prepulse (75, 80, or 85 dB) + pulse stimulation, prepulse (75, 80, or 85 dB) alone, and no stimulus. Testing was completed within 40 min. The results, designated PP75, PP80, and PP85, were calculated automatically by the system software. The percentage PPI induced by each prepulse intensity was calculated as [1 − (startle amplitude on prepulse trial/startle amplitude on pulse alone)] × 100% [46].

### Immunohistochemical (IHC) study

We chose 23 animals (13 males as cases; 10 males as controls) at PN60 and 23 animals (15 males as cases; 8 males as controls) at PN40 for the IHC study to observe the morphological changes in the activated microglia and astrocytes in the prefrontal cortex (PFC) and hippocampus (HC) of the offspring brains. The animals were anesthetized with isoflurane and perfused with 4% paraformaldehyde. They were postfixed overnight and cryprotected in 30% sucrose solution for 48 h at 4 °C. Serial sections of the brain were cut to 20 μm with a cryostat microtome (Leica CM1850). Six discontinuous slices of each region of each brain were selected to count the densities of microglia and astrocytes under an optical microscope. The slices were rinsed in PBS, and stored at − 20 °C until further processing. For immunostaining, the slices were rinsed three times for 10 min in PBS. Blocking was done in 5% normal serum for 1 h at room temperature. The following primary antibodies were used: goat anti-IBA1 primary antibody (1:500; Abcam, Cambridge, UK), rabbit anti-GFAP (1:300, Boster, Wu Han, China). They were incubated overnight at room temperature. After three washes with PBS (2 min each), the sections were incubated for 1 h with the biotinylated secondary antibodies diluted 1:500. Sections were washed again three times for 2 min in PBS and incubated with the strept avidin–biotin complex for 1 h. Then rinsed again four times for 5 min in PBS, dehydrated, and coverslipped with Eukitt (Kindler, Freiburg, Germany). The GFAP-labeled slices were counterstained with 0.25% cresyl violet according to standard protocols. After staining, the sections were dehydrated through an alcohol series, cleared with xylene, and coverslipped with Eukitt.

### Enzyme-linked immunosorbent assays (ELISAs)

Three pregnant rats were humanely killed about 3 h after the administration of Poly I:C or vehicle at G9. And we respectively chose 15 animals (8 males as cases; 7 males as controls) at PN60 and at PN40 for the ELISA study to observe the expression level changes in the prefrontal cortex (PFC) and hippocampus (HC) of the offspring brains. The plasma from their heart blood was isolated within 30 min of collection by centrifugation for 20 min at 1000×*g*, and the samples were then stored as aliquots at ≤ − 70 °C before their IL-1β, IL-6, and TNF-α concentrations were measured. Some offspring of the animals in each group were humanely killed at both ages, and tissue homogenates (diluted 1:10 in 0.9% saline) of the target brain regions were separated to measure the concentrations of IL-1β, IL-6, and TNF-α. The subsequent procedures were all performed with highly sensitive ELISAs from R&D Systems (Minneapolis, Minnesota, USA), according to the manufacturer’s instructions.

### Statistical analyses

The data are presented as means ± standard deviations and were analyzed with SPSS 20.0 (SPSS Inc., Chicago, IL, USA). Student’s *t* test was used for the data analysis, according to the nature of the data. All results are two-sided, and the level of significance is α = 0.05.

## Results

### PPI test

**Fig. 1:**
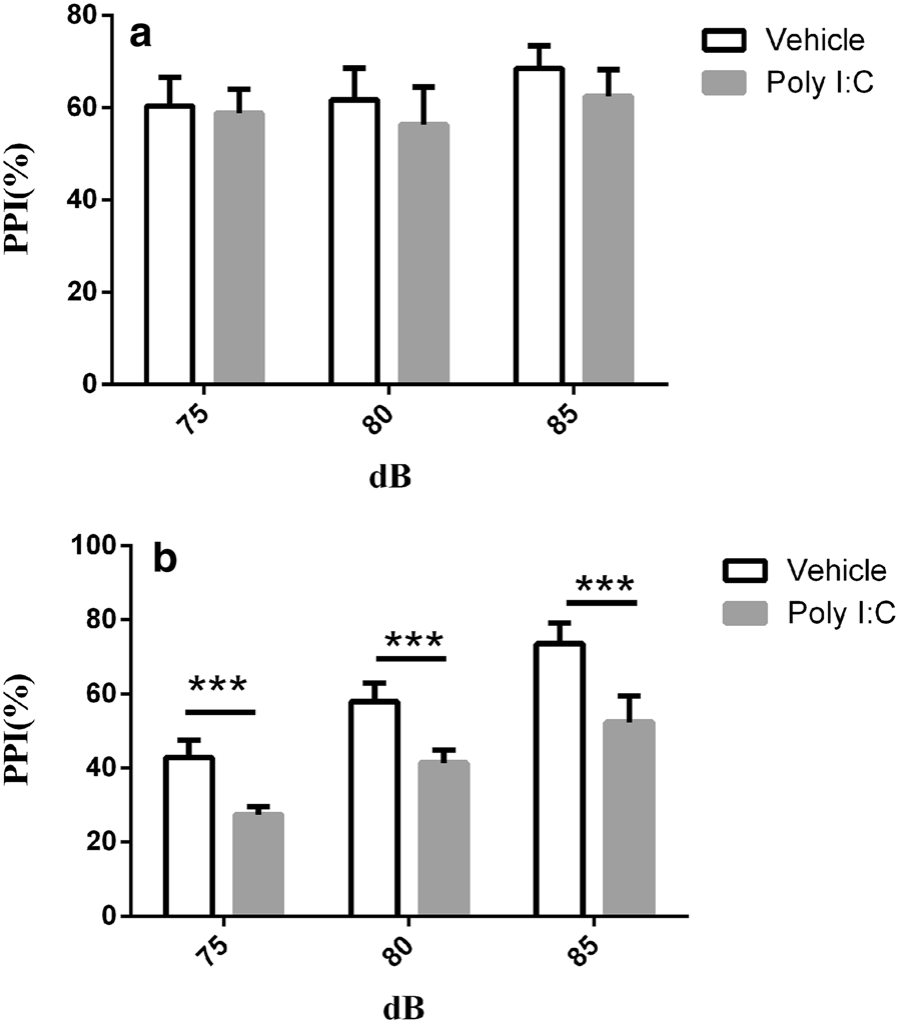
Effects of early maternal immune activation on prepulse inhibition in rat offspring. Data are shown as mean ± SEM. N = 10 for each group. **a** Maternal immune activation produced no significant differences in any dB group (70, 75, or 80 dB) in adolescence. **b** Prepulse inhibition defects were observed in all dB groups (70, 75, and 80 dB) in adult offspring of dams exposed to Poly I:C at gestational day 9. ***P < 0.001

Figure 1 shows the effects of early prenatal Poly I:C administration on PPI deficits in the adolescent and adult rat offspring. The adult offspring displayed significant inhibition (all *P* < 0.01) compared with the control offspring in all the dB groups (72, 74, and 78 dB), whereas the adolescent offspring did not. These results confirm that MIA in the first trimester can induce disrupted PPI after puberty in rats, which is similar to the findings in schizophrenia patients.

### Analysis of microglial markers

**Fig. 2:**
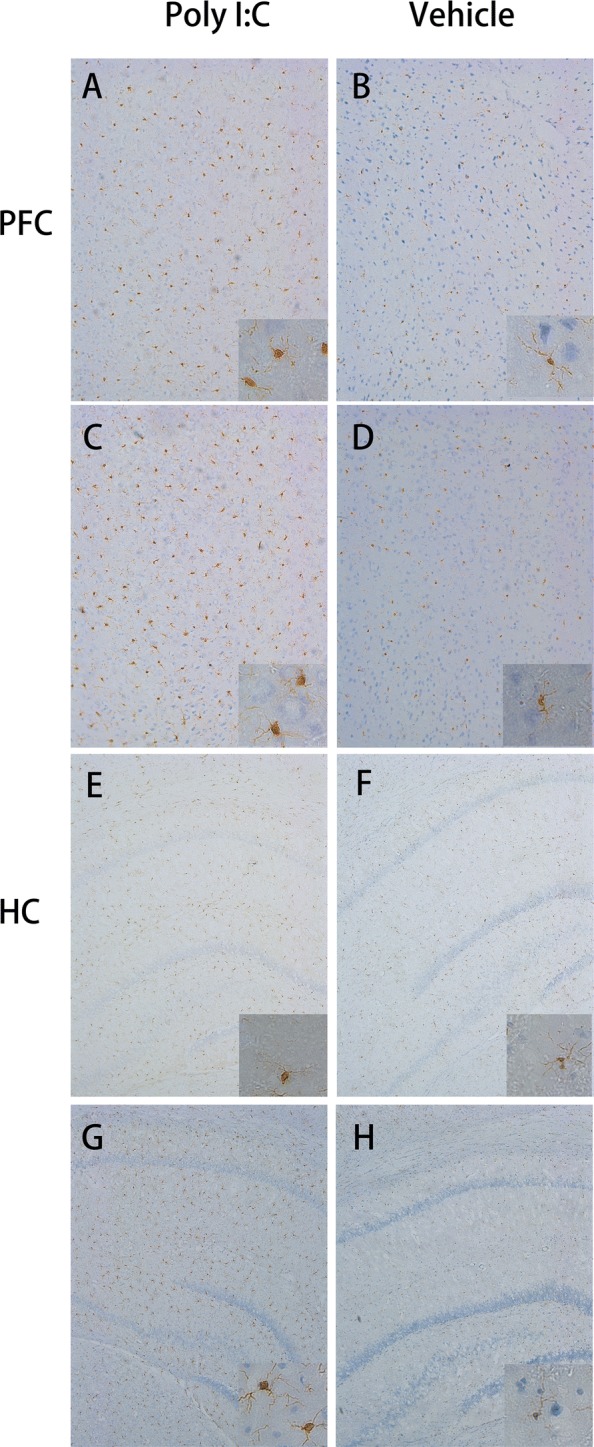
Effects of early maternal immune activation on microglial activation in the prefrontal cortex (PFC) and hippocampus (HC) regions of offspring. **A**–**D** Representative images of immunohistochemical staining for ionized calcium-binding adapter molecule 1 (IBA1) in PFC of rats after early prenatal treatment with Poly I:C (**A**, **C**) or vehicle (**B**, **D**). Row 1 and row 2: During puberty (row 1) and adulthood (row 2), IBA1-positive cells in the PFC were with round cell bodies and reduced arborizationin the offspring of rats exposed to Poly I:C at gestational day 9; n = 9 for Poly I:C group, n = 7 for vehicle group. **E**–**H** Representative images of immunohistochemical staining for IBA1 in HC of rats after early prenatal treatment with Poly I:C (**E**–**G**) or vehicle (**F**–**H**); n = 13 for Poly I:C group, n = 10 for vehicle group. Row 3 and row 4: IBA1-positive cells in the HC were also with round cell bodies and reduced arborization during puberty (row 3) and adulthood (row 4) in the offspring of rats exposed to Poly I:C at GD9 (×10)

**Fig. 3:**
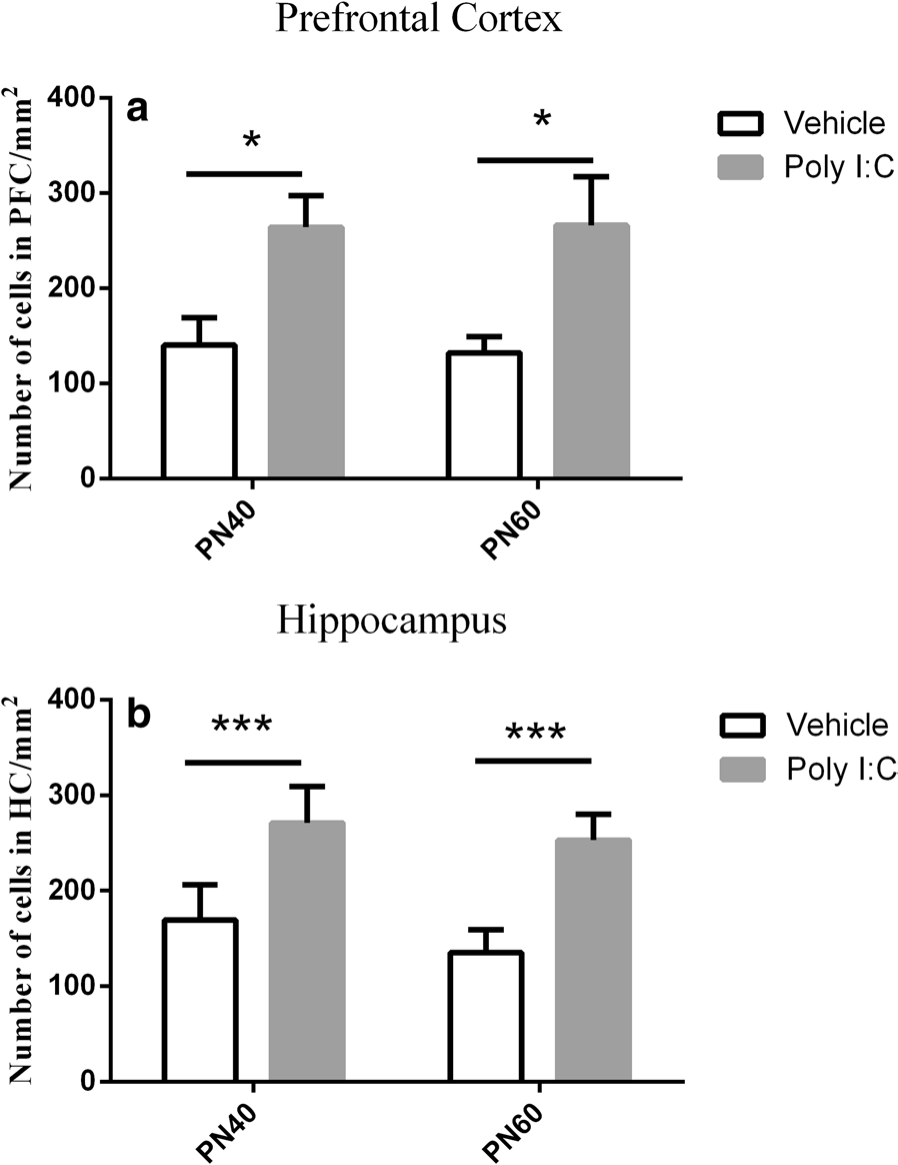
Comparison of number of ionized calcium-binding adapter molecule 1 (IBA1)-immunopositive cells in prefrontal cortex (**a**), hippocampus (**b**) between pubescent and adult offspring based on Student’s *t* test. The data is shown as mean ± SEM. *P < 0.05, ***P < 0.001

In both the PFC and HC, more activated microglia were observed in the Poly I:C offspring at the age of either 40 or 60 days than in the corresponding controls. Figure 2 clearly suggests increased numbers of microglia in both the regions examined in the Poly I:C-treated offspring. The morphology of the microglia in the two target regions also clearly differs from that in the vehicle-treated offspring. In detail, the activated microglia in the offspring of the MIA rats were characterized by enlarged cell bodies, with retracted and thickened processes, which differed from the microglia in the quiescent state observed in vehicle-treated offspring, which had round cell bodies and thin processes, with simple ramifications. We counted number of Iba1-immunopositive cells in the PFC and HC for statistical comparison. As shown in Fig. 3a, b, two-sided Student’s *t* test analysis revealed that the number of Iba1-immunopositive cells in these brain regions of Poly I:C-treated group was significantly higher when compared with control group (all *P *< 0.001).

### Analysis of astrocyte markers

**Fig. 4:**
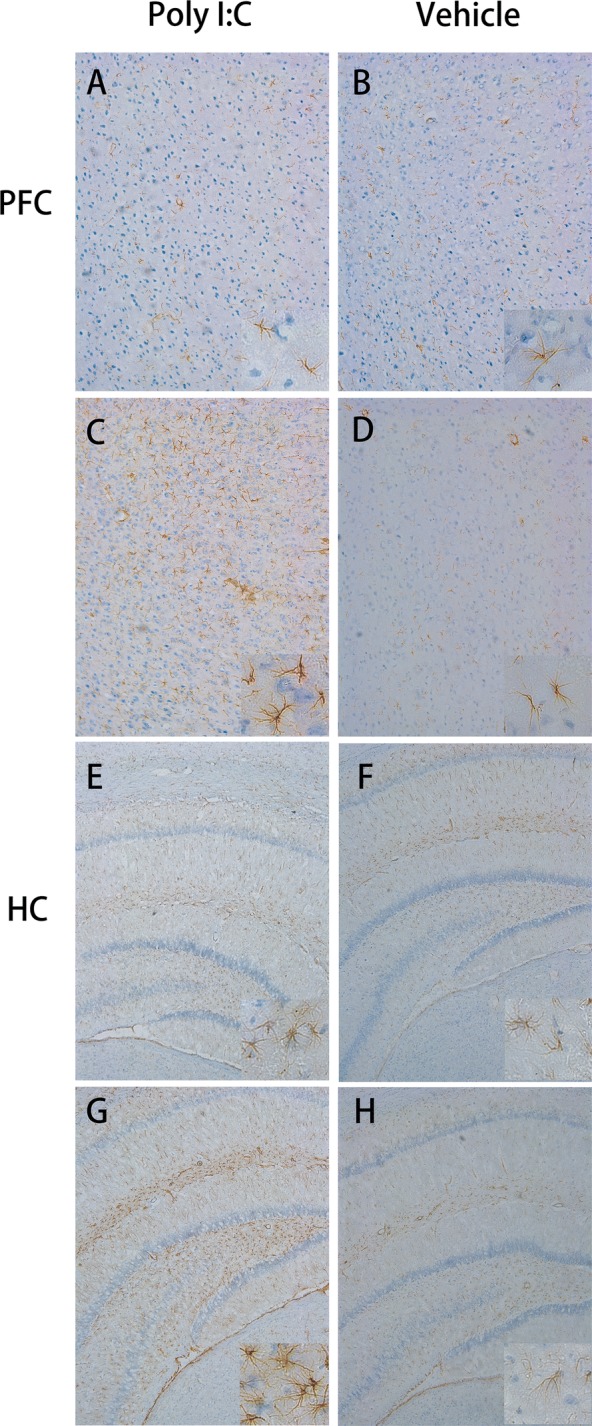
Effects of early maternal immune activation on astrocytes in the prefrontal cortex and hippocampus regions of offspring. **A**–**D** Representative images of immunohistochemical staining for glial fibrillary acidic protein (GFAP) in the PFC of rats after early prenatal treatment with Poly I:C (**A**, **C**) or vehicle (**B**, **D**); n = 15 for Poly I:C group; n = 8 for vehicle group. During puberty (row 1, row 3), there were no significant differences in astrocytes in the two target brain regions. **E**–**H** Representative images of immunohistochemical staining for GFAP in the HC of rats after early prenatal treatment with Poly I:C or vehicle; n = 9 for Poly I:C group; n = 7 for vehicle group. Row 2 and row 4: during adulthood, GFAP-positive cells with a hypertrophic morphology were observed in the PFC and HC of offspring of mice exposed to Poly I:C at gestational day 9 (×10)

**Fig. 5:**
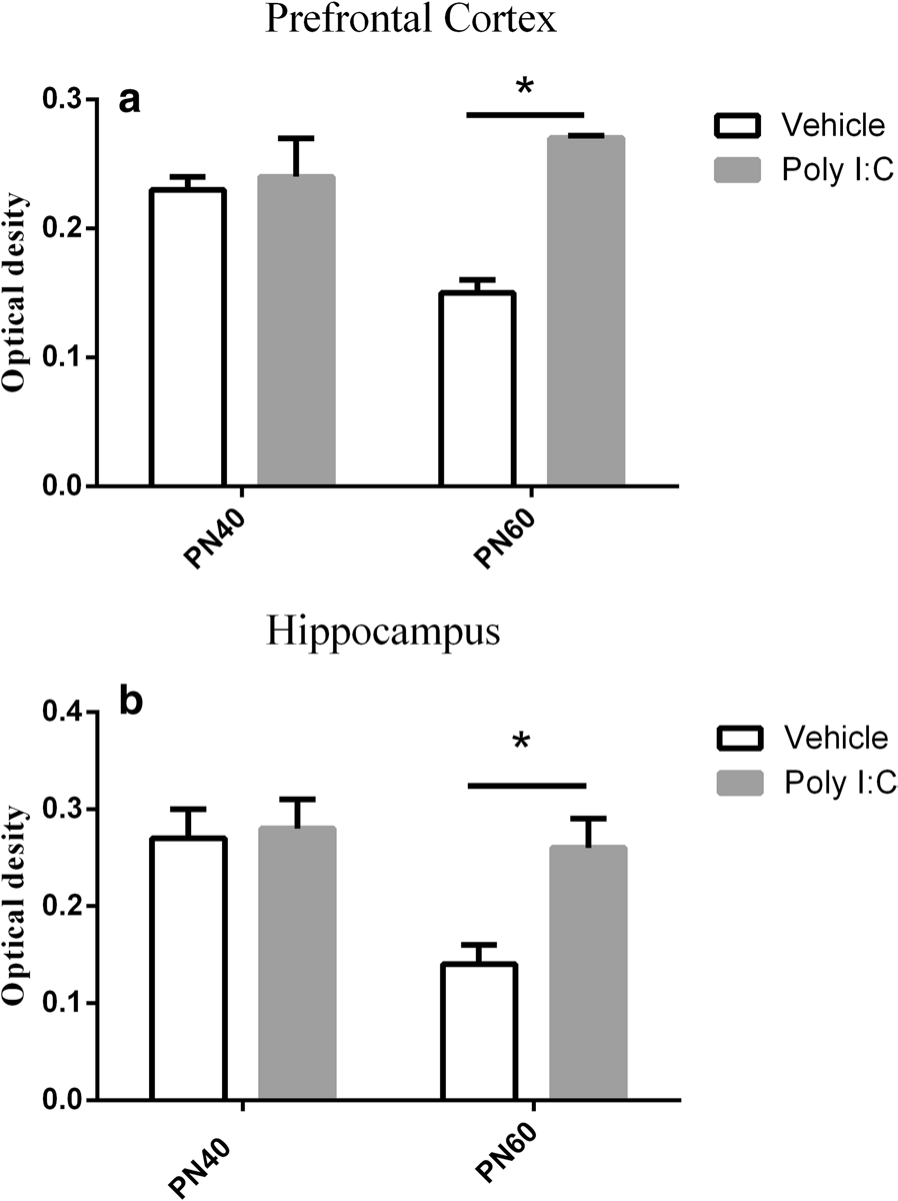
Comparison of optical density of glial fibrillary acidic protein (GFAP)-immunopositive cells in prefrontal cortex (**a**), hippocampus (**b**) between pubescent and adult offspring based on Student’s *t* test. The data is shown as mean ± SEM. *P < 0.05

Figure 4 shows the age-dependent status of the astrocytes in the prenatally Poly I:C-treated rats. As shown in Fig. 5, the astrocytes did not differ significantly between the two groups on PN40, but on PN60, the optical density of glial fibrillary acidic protein-positive astrocytes in these brain regions of the MIA rat offspring was significantly higher when compared with control group (all *P *< 0.001). Besides, the hypertrophic astrocyte morphology had increased in the MIA rat offspring.

### Analysis of proinflammatory cytokines

**Fig. 6:**
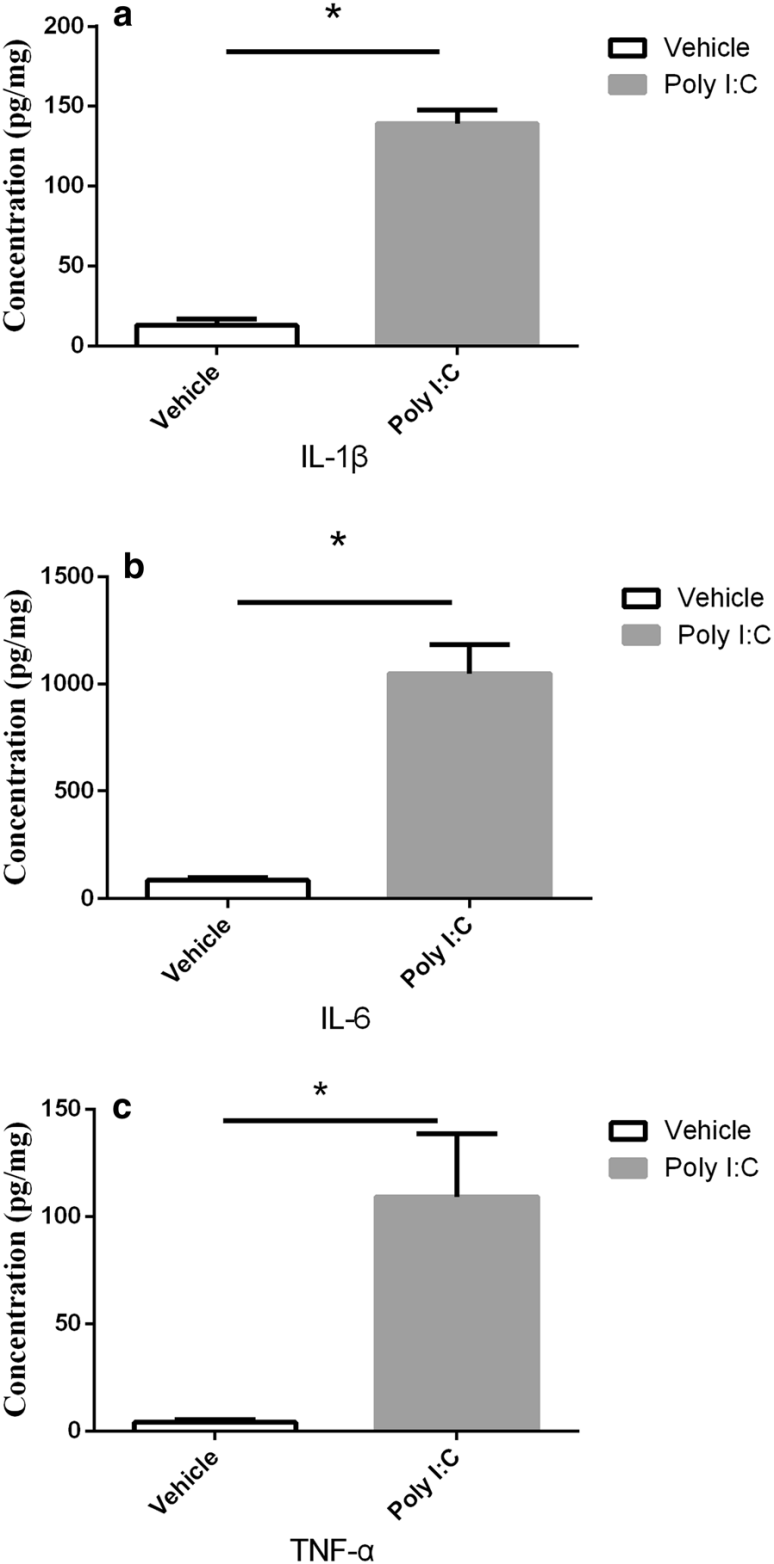
Cytokine (IL-1β, IL-6, and TNF-α) Levels of pregnant rats treated with Poly I:C or NaCl on gestational day 9. All of these cytokines elevated in the blood of Poly I:C rats. Data are shown as mean ± SEM; n = 3 for each group. **a** Concentrations (pg/mg) of IL-1β; **b** concentrations (pg/mg) of IL-6; **c** concentrations (pg/mg) of TNF-α. *P < 0.05

**Fig. 7:**
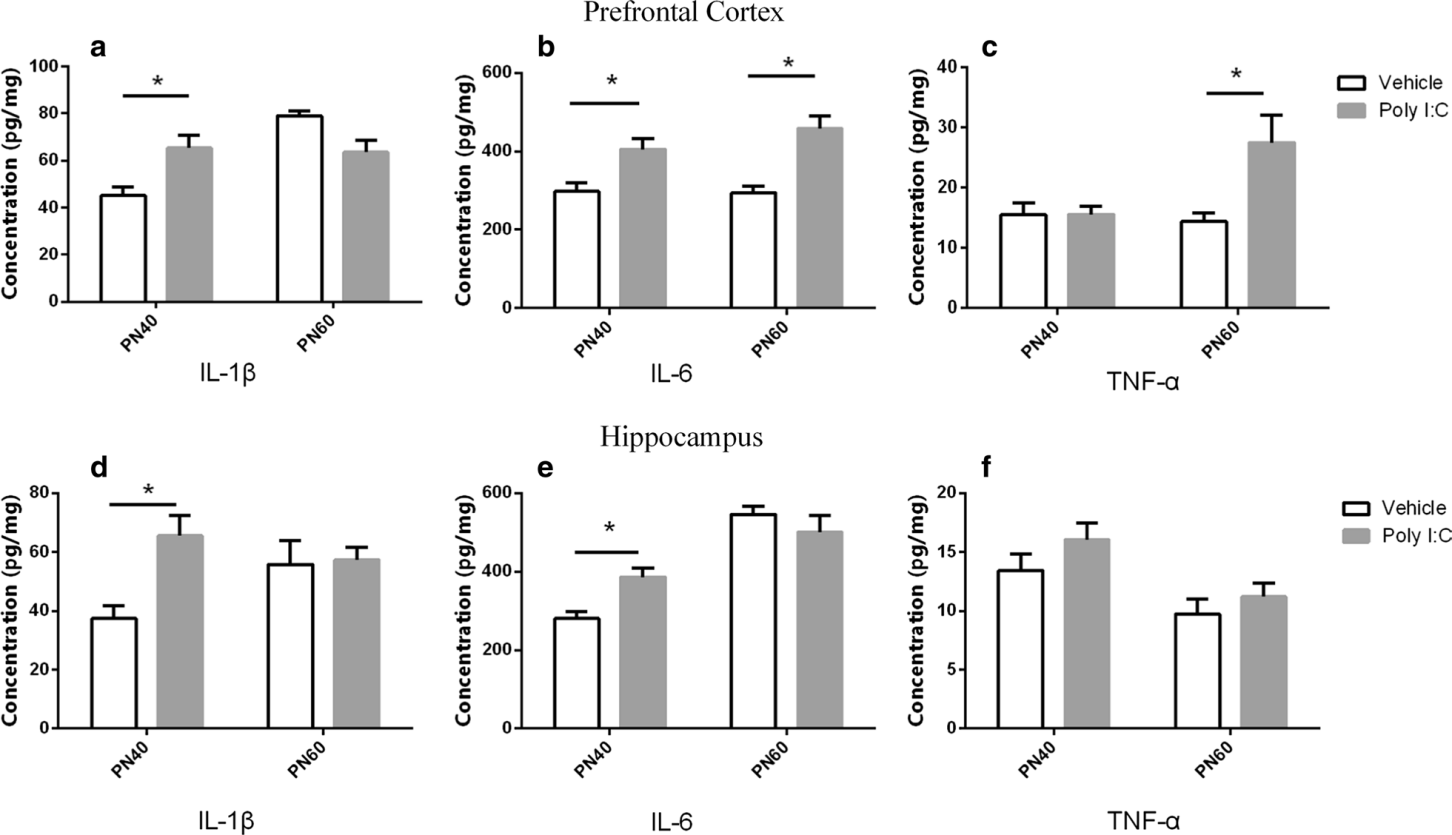
Effects of early maternal immune activation (MIA) on cytokines (IL-1β, IL-6, and TNF-α) in the prefrontal cortex (PFC) and hippocampus (HC) regions of MIA rat offspring during adolescence and adulthood. Overall, most of these cytokines behaved in region- and age-related manners. Data are shown as mean ± SEM; n = 8 for Poly I:C group, n = 7 for vehicle group. **a**–**c** Concentrations (pg/mg) of IL-1β, IL-6, and TNF-α in the PFC during adolescence and adulthood. **d**–**f** Concentrations (pg/mg) of IL-1β, IL-6, and TNF-α in the HC during adolescence and adulthood. *P < 0.05

As shown in Fig. 6, expression of all three cytokines (IL-1β, IL-6 and TNF-α) have elevated in pregnant rats administrated with Poly I:C, which have validated our successful injection. Additionally, proinflammatory cytokine levels changed differently in the two groups of rat offspring at different stages, as shown in Fig. 7. The Poly I:C-treated pubescent offspring showed significantly higher concentrations of IL-1β and IL-6 than the controls in both the PFC and HC. In the young adult offspring, no significant differences were detected in the HC, whereas in the PFC, the levels of TNF-α and IL-6 were elevated. The Poly I:C treatment did not affect the concentrations of TNF-α in the HC in either the pubescent or young adult rats.

## Discussion

Schizophrenia typically emerges in late adolescence or early adulthood. Studies in adulthood can help us understand the characteristics of this disease, whereas research that focuses on adolescence may provide targets for preventive strategies. In this study, we investigated the functional profiles of microglia, astrocytes, and cytokines with regard to the progressive behavioral changes that occurred from puberty to young adulthood in a Poly I:C-induced rat model of MIA.

Sensorimotor gating is one of the core biological features of schizophrenia [47]. Auditory sensory gating can be detected with the PPI test in both patients and animal models. Our data confirm that the administration of Poly I:C in the first trimester causes PPI defects in early adulthood. This result is consistent with other research with Poly I:C-induced animal models of infection during pregnancy and the clinical characteristics of schizophrenia [48]. The initial epidemiological data suggested that maternal viral challenge during mid or late human pregnancy enhances the risk of schizophrenia in the adult offspring [46]. Therefore, many preclinical animal studies have been performed to confirm this finding. Several studies then demonstrated that immune stimulation during early gestation may also be critical [41]. However, preclinical information is limited, especially in rats. Our data provide new evidence of this phenomenon.

We found that all three components involved in the neural immune process (microglia, astrocytes, and cytokines) were altered in the Poly I:C-treated offspring during development and that these changes displayed distinct patterns. Our results suggest that at PN40, the microglia were already activated, and this activation persisted into early adulthood. This is consistent with a previous study in the neonatal offspring of Poly I:C-challenged rats [49]. However, two published studies of mice reported that microglia were only activated at PN30, not PN100 [48, 50], and Zhu et al. noted that the numbers of IBA1-positive microglia in the HC and cerebral cortex were increased in the adult (PN 62) offspring of Poly I:C-treated rats [51]. A possible interpretation of these discrepancies is that microglial activation varies with the animal strain, the time of stimulation, the brain region tested, and the age of the offspring examined. Therefore, as the major immune cell population in the brain, microglia are believed to participate in the pathogenesis of schizophrenia, although the time of their activation remains controversial.

Consistent with the behavioral findings, astrocytes were only activated in early adulthood in the MIA rat offspring, which may indicate a crucial link between behavioral abnormalities and astrocyte activation. Although studies have reported no significant changes in the astrocytes of patients with schizophrenia [27], the samples examined were usually highly heterogeneous, with various confounding factors, such as antipsychotic medication and illness stage. In our experiment, we documented astrogliosis in the young adult offspring of MIA rats and compared it with that in the controls. A recent study detected reactive astrocytes in subsets of people with schizophrenia with high levels of inflammatory markers in the PFC [52]. Therefore, we speculate that inflammation may be a link between astrocytes and PPI, and this warrants further investigation.

It is noteworthy that the offspring of rats with early maternal MIA displayed more-elevated cytokines in adolescence than in adulthood. As well as being age-related, these cytokine changes were also region-specific. In PFC, the majority of cytokines were elevated in adolescence, and only elevated IL-6 persisted into adulthood. No changes in TNF-α levels were observed until adulthood. The changes in the HC differed from those in the PFC, and the levels of most cytokines (including IL-6) were only increased during puberty, and not in adulthood. Notably, IL-6 is an important cytokine in cerebral function [53, 54] and is associated with schizophrenia [55]. Smith and his colleagues found that the adult offspring of mice prenatally administered IL-6 displayed schizophrenia-like deficits, and that the coadministration of an anti-IL-6 antibody to the mouse model prevented the aberrant phenotype [56].

It has been suggested that the injection of Poly I:C into pregnant rats alters the brain cytokines of their offspring, based on the facts that neuroinflammation and cytokines are altered in the brains and cerebrospinal fluid of schizophrenia patients [57, 58]. At the same time, it posed an interesting question as to how cytokine cause schizophrenia. One study shown that cytokine proteins inhibit hippocampal neurogenesis and the level of microglia was negatively correlated with neurogenesis [59]. Besides, neuroinflammation may influence the glutamatergic and dopamine system [60, 61].

Clinical data predict that inflammatory cytokines increase progressively in the brains of MIA offspring, but there is little detailed information on the changes at different stages of development, especially in adolescence. A recent study by has provided some evidence for this, and our data are consistent with it [14]. However, previous studies have lacked the corresponding behavioral information. More importantly, our findings may be the first to relate the status of astrocytes and PPI.

While our study has explored two key time points of schizophrenia, it still has some limitation. First, we documented that evident neuroinflammation has already existed at adolescence, but what is the situation with earlier developmental stages? In the future, we will provide more information about neuro-immune process from fetal to late adult brains in animal models. Second, we found the same turning point of both astrocyte and PPI, which needs further precise investigation in if specific status of astrocyte contribute to specific behavioral abnormalities in offspring.

## Conclusion

In summary, early maternal infection can induce immune activation, causing increases in activated gliosis and proinflammatory cytokines in the offspring at two key ages, especially periadolescence. This may be a more important stage than young adulthood for the pathogenesis of schizophrenia, leading to abnormal behavior in the adult stage. From this perspective, interventions that regulate immunological activity in an early developmental stage may be important and offer promising therapeutic strategies for schizophrenia.

## Abbreviations

MIA: maternal immune activation
PPI: prepulse inhibition
PN: postnatal day
Poly I: C: polyriboinosinic–polyribocytidylic acid
PFC: prefrontal cortex
HC: hippocampus
Iba1: ionized calcium-binding adapter molecule 1
GFAP: glial fibrillary acidic protein
CNS: central nervous system
IL: interleukin
TNF-α: tumor necrosis factor α
TGF-β: transforming growth factor β
IFN: interferon
GD: gestational day
IHC: immunohistochemical

## References

[1] Tandon R, Keshavan MS, Nasrallah HA (2008). Schizophrenia, “Just the Facts”: what we know in 2008 part 1: overview. Schizophr Res.

[2] Hall J (2015). Genetic risk for schizophrenia: convergence on synaptic pathways involved in plasticity. Biol Psychiat.

[3] Depino AM (2018). Perinatal inflammation and adult psychopathology: from preclinical models to humans. Semin Cell Dev Biol.

[4] Brown AS, Derkits EJ (2010). Prenatal infection and schizophrenia: a review of epidemiologic and translational studies. Am J Psychiatry..

[5] Torrey EF, Bartko JJ, Yolken RH (2012). *Toxoplasma gondii* and other risk factors for schizophrenia: an update. Schizophrenia Bull..

[6] Mednick SA (1998). Adult schizophrenia following prenatal exposure to an influenza epidemic. Arch Gen Psychiatry..

[7] Winter C (2009). Prenatal immune activation leads to multiple changes in basal neurotransmitter levels in the adult brain: implications for brain disorders of neurodevelopmental origin such as schizophrenia. Int J Neuropsychopharmacol.

[8] Kirkpatrick B, Miller BJ (2013). Inflammation and schizophrenia. Schizophrenia Bull..

[9] Trepanier MO (2016). Postmortem evidence of cerebral inflammation in schizophrenia: a systematic review. Mol Psychiatry..

[10] Kahn RS, Sommer IE (2015). The neurobiology and treatment of first-episode schizophrenia. Mol Psychiatry..

[11] Van Kesteren CFMG (2017). Immune involvement in the pathogenesis of schizophrenia: a meta-analysis on postmortem brain studies. Transl Psychiatry..

[12] Arion D (2007). Molecular evidence for increased expression of genes related to immune and chaperone function in the prefrontal cortex in schizophrenia. Biol Psychiatry..

[13] Monji A (2013). Neuroinflammation in schizophrenia especially focused on the role of microglia. Progress Neuro Psychopharmacol Biol Psychiatry..

[14] Garay PA (2013). Maternal immune activation causes age- and region-specific changes in brain cytokines in offspring throughout development. Brain Behav Immunity..

[15] Miller BJ (2013). Prenatal inflammation and neurodevelopment in schizophrenia: a review of human studies. Prog Neuropsychopharmacol Biol Psychiatry.

[16] Söderlund J (2009). Activation of brain interleukin-1beta in schizophrenia. Mol Psychiatry..

[17] Takahashi N, Sakurai T (2013). Roles of glial cells in schizophrenia: possible targets for therapeutic approaches. Neurobiol Dis..

[18] Schwarz JM, Bilbo SD (2012). Sex, glia, and development: interactions in health and disease. Horm Behav.

[19] Bayer TA (1999). Evidence for activation of microglia in patients with psychiatric illnesses. Neurosci Lett.

[20] Fillman SG (2013). Increased inflammatory markers identified in the dorsolateral prefrontal cortex of individuals with schizophrenia. Mol Psychiatry..

[21] Radewicz K (2000). Increase in HLA-DR immunoreactive microglia in frontal and temporal cortex of chronic schizophrenics. J Neuropathol Exp Neurol.

[22] Barley K, Dracheva S, Byne W (2009). Subcortical oligodendrocyte- and astrocyte-associated gene expression in subjects with schizophrenia, major depression and bipolar disorder. Schizophrenia Res..

[23] Feresten AH (2013). Increased expression of glial fibrillary acidic protein in prefrontal cortex in psychotic illness. Schizophr Res.

[24] Arnold SE (1998). Absence of neurodegeneration and neural injury in the cerebral cortex in a sample of elderly patients with schizophrenia. Arch Gen Psychiatry..

[25] Kano S (2011). Altered MHC class I expression in dorsolateral prefrontal cortex of nonsmoker patients with schizophrenia. Neurosci Res.

[26] Steffek AE (2008). Cortical expression of glial fibrillary acidic protein and glutamine synthetase is decreased in schizophrenia. Schizophr Res.

[27] Webster MJ (2005). Glial fibrillary acidic protein mRNA levels in the cingulate cortex of individuals with depression, bipolar disorder and schizophrenia. Neuroscience.

[28] Stertz L, Magalhães PV, Kapczinski F (2013). Is bipolar disorder an inflammatory condition? The relevance of microglial activation. Curr Opin Psychiatry..

[29] Réus GZ (2015). The role of inflammation and microglial activation in the pathophysiology of psychiatric disorders. Neuroscience.

[30] Majumder S, Zhou ZH, Ransohoff RM (1996). Transcriptional regulation of chemokine gene expression in astrocytes. J Neurosci Res.

[31] Farina S (2007). Astrocytes are active players in cerebral innate immunity. Trends Immunol..

[32] Meyer U (2005). Towards an immuno-precipitated neurodevelopmental animal model of schizophrenia. Neurosci Biobehav Rev.

[33] Shi L (2003). Maternal influenza infection causes marked behavioral and pharmacological changes in the offspring. J Neurosci Off J Soc Neurosci.

[34] Osborne AL, Solowij N, Babic I (2017). Improved social interaction, recognition and working memory with cannabidiol treatment in a prenatal infection (poly I:C) rat model. Neuropsychopharmacology..

[35] Dickerson DD (2013). Aberrant neural synchrony in the maternal immune activation model: using translatable measures to explore targeted interventions. Front Behav Neurosci..

[36] Piontkewitz Y, Bernstein HG, Dobrowolny H (2012). Effects of risperidone treatment in adolescence on hippocampal neurogenesis, parvalbumin expression, and vascularization following prenatal immune activation in rats. Brain Behav Immun.

[37] Hadar R (2015). Using a maternal immune stimulation model of schizophrenia to study behavioral and neurobiological alterations over the developmental course. Schizophr Res.

[38] Piontkewitz Y, Arad M, Weiner I (2012). Tracing the development of psychosis and its prevention: what can be learned from animal models. Neuropharmacology..

[39] Piontkewitz Y, Arad M, Weiner I (2011). Abnormal trajectories of neurodevelopment and behavior following in utero insult in the rat. Biol Psychiatry..

[40] Meyer U (2010). Evaluating early preventive antipsychotic and antidepressant drug treatment in an infection-based neurodevelopmental mouse model of schizophrenia. Schizophr Bull.

[41] Sørensen HJ (2009). Association between prenatal exposure to bacterial infection and risk of schizophrenia. Schizophr Bull.

[42] Meyer U (2014). Prenatal Poly I: C exposure and other developmental immune activation models in rodent systems. Biol Psychiat.

[43] Sullivan R (2006). The international society for developmental psychobiology annual meeting symposium: impact of early life experiences on brain and behavioral development. Developmental Psychobiology..

[44] Meyer U, Feldon J (2012). To Poly I: C or not to Poly I:C: advancing preclinical schizophrenia research through the use of prenatal immune activation models. Neuropharmacology..

[45] Batinić B (2016). Lipopolysaccharide exposure during late embryogenesis results in diminished locomotor activity and amphetamine response in females and spatial cognition impairment in males in adult, but not adolescent rat offspring. Behav Brain Res.

[46] Braff DL, Geyer MA, Swerdlow NR (2001). Human studies of prepulse inhibition of startle: normal subjects, patient groups, and pharmacological studies. Psychopharmacology.

[47] Cromwell HC (2008). Sensory gating: a translational effort from basic to clinical science. Clin EEG Neurosci.

[48] Eßlinger M (2016). Schizophrenia associated sensory gating deficits develop after adolescent microglia activation. Brain Behav Immun.

[49] Ribeiro BM (2013). Evidences for a progressive microglial activation and increase in iNOS expression in rats submitted to a neurodevelopmental model of schizophrenia: reversal by clozapine. Schizophr Res.

[50] Manitz MP (2013). The role of microglia during life span in neuropsychiatric disease—an animal study. Schizophr Res.

[51] Zhu F (2014). Minocycline alleviates behavioral deficits and inhibits microglial activation in the offspring of pregnant mice after administration of polyriboinosinic–polyribocytidilic acid. Psychiatry Res.

[52] Catts VS (2014). Increased expression of astrocyte markers in schizophrenia: association with neuroinflammation. Aust N Z J Psychiatry.

[53] Samuelsson AM (2006). Prenatal exposure to interleukin-6 results in inflammatory neurodegeneration in hippocampus with NMDA/GABA(A) dysregulation and impaired spatial learning. Am J Physiol Regul Integr Comp Physiol.

[54] Stolp HB (2013). Neuropoietic cytokines in normal brain development and neurodevelopmental disorders. Mol Cell Neurosci.

[55] Wei H (2011). IL-6 is increased in the cerebellum of autistic brain and alters neural cell adhesion, migration and synaptic formation. J Neuroinflamn..

[56] Smith SE (2007). Maternal immune activation alters fetal brain development through interleukin-6. J Neurosci.

[57] Yang L (2011). Elevated plasma cytokines in autism spectrum disorders provide evidence of immune dysfunction and are associated with impaired behavioral outcome. Brain Behav Immun.

[58] Ashwood P (2011). Associations of impaired behaviors with elevated plasma chemokines in autism spectrum disorders. J Neuroimmunol.

[59] Monje ML, Toda H, Palmer TD (2003). Inflammatory blockade restores adult hippocampal neurogenesis. Science.

[60] Muller N, Myint AM, Schwarz MJ (2011). Kynurenine pathway in schizophrenia: pathophysiological and therapeutic aspects. Curr Pharm Des.

[61] Erhardt S, Engberg G (2002). Increased phasic activity of dopaminergic neurones in the rat ventral tegmental area following pharmacologically elevated levels of endogenous kynurenic acid. Acta Physiol Scand.

